# Effects of Diet and Exercise Lifestyle Interventions on Physical and Psychological Health in Breast Cancer Survivors: A Systematic Review

**DOI:** 10.3390/nu18111815

**Published:** 2026-06-04

**Authors:** Nuria Asencio-Mas, Maria Martínez-Olcina, Belén Leyva-Vela, Manuel Vicente-Martínez, Yolanda Nadal-Nicolás, Jose Manuel Garcia-De Frutos, Alejandro Martínez-Rodríguez

**Affiliations:** 1 Department of Analytical Chemistry, Nutrition and Food Science, University of Alicante, 03690 Alicante, Spain; nuria.asencio@ua.es (N.A.-M.); bmlv1@alu.ua.es (B.L.-V.); mvmr2@alu.ua.es (M.V.-M.); 2 Faculty of Health Sciences, International University of La Rioja (UNIR), 26006 Logroño, Spain; maria.martinezolcina@unir.net; 3 European Institute of Exercise and Health, 03202 Elche, Spain; 4 Faculty of Medicine, Miguel Hernández University of Elche, 03202 Elche, Spain; yolanda.nadal@umh.es; 5 Faculty of Sports Science, Catholic University of Murcia, 30107 Murcia, Spain; jmgarcia887@ucam.edu

**Keywords:** breast cancer, exercise, nutrition, body composition, quality of life

## Abstract

Breast cancer survivors frequently experience adverse changes in body composition, cardiometabolic biomarkers, functional capacity and quality of life that may worsen long-term prognosis, yet the comparative effectiveness of lifestyle interventions across delivery formats and supervision levels remains unclear. **Background/Objectives:** This systematic review assessed the effects of structured diet and exercise interventions on body composition, metabolic and inflammatory biomarkers, functional capacity, dietary habits and quality of life in breast cancer survivors. **Methods:** Following PRISMA guidelines, Cochrane, PubMed, Scopus and Web of Science were searched for randomized controlled trials and quasi-experimental studies published in English between 2016 and 2026. Risk of bias was assessed with RoB 2 and ROBINS-I and certainty of evidence with GRADE. **Results:** Of 1413 records, 15 studies (11 RCTs; mean age 46–60 years; mostly overweight or obese post-treatment women) met the inclusion criteria; twelve interventions were supervised and three home-based or web-based. Within the assessed domains, many studies reported significant improvements in body composition, quality of life and metabolic or inflammatory biomarkers. Effects were larger in multimodal supervised programs combining caloric restriction with moderate-to-vigorous aerobic plus resistance training (5–8% weight loss; 19–29% visceral fat reduction; improved insulin, IGF-1, leptin, adiponectin and EORTC QLQ-C30 scores), whereas digital or low-intensity interventions produced smaller, less uniform objective effects despite improving dietary behaviors. GRADE certainty ranged from very low to moderate–high. **Conclusions:** Multimodal supervised programs offer the most robust benefits; digital formats require additional supervision. Standardized protocols and longer follow-up are needed.

## 1. Introduction

Breast cancer is the most common malignant tumor in women worldwide. Thanks to advances in early diagnosis and treatment, survival rates have risen steadily over the past few decades. As a result, scientific and clinical attention has shifted toward the challenges of survivorship, including tumor recurrence, long-term treatment side effects, and the development of secondary primary tumors.

The available scientific evidence indicates that being overweight at the time of diagnosis and weight gain following treatment are associated with a poorer prognosis, a higher risk of recurrence, and higher overall mortality among breast cancer survivors. In this context, lifestyle interventions combining dietary modifications and physical exercise have shown beneficial effects on body weight, waist circumference, and various metabolic biomarkers related to long-term prognosis [1,2,3]. These changes not only affect body composition but may also influence key biological mediators such as insulin, leptin, and various inflammatory markers.

In particular, elevated insulin levels and abnormalities in the insulin-like growth factor (IGF-1) axis have been linked to an increased risk of recurrence and mortality in breast cancer survivors. Randomized clinical trials have shown that aerobic or combined exercise programs can significantly reduce insulin and growth factor levels [4,5], suggesting that physical activity may modulate biological mechanisms potentially involved in tumor progression.

Beyond metabolic mechanisms, low-grade chronic inflammation and immune dysfunction have also been implicated in tumor progression and health outcomes during cancer survival. Strength training has shown beneficial effects on inflammatory markers and on the activity of immune cells such as NK and NKT cells, which play a significant role in tumor surveillance and in the interaction between innate and adaptive immunity [6]. These findings support the hypothesis that physical exercise may provide benefits that go beyond improving physical fitness.

From a functional and cardiovascular perspective, regular physical activity improves hemodynamic efficiency and reduces myocardial workload during submaximal exercise, which is associated with lower levels of fatigue and better physiological adaptation [7]. Furthermore, higher levels of cardiorespiratory fitness and physical activity have been linked to better cognitive performance in breast cancer survivors [8], a relevant finding given that persistent fatigue and cognitive impairment can significantly affect quality of life and daily functioning.

The psychological component is another key factor in cancer survival. Cancer-related fatigue, anxiety, depression, and sleep disturbances can act as barriers to adopting and maintaining healthy lifestyle habits, while also contributing to persistent systemic inflammation. Structured exercise interventions have been shown to reduce fatigue and modulate inflammatory cytokines involved in its pathophysiology [9]. Similarly, self-care programs based on exercise and nutrition have demonstrated improvements in quality of life, physical functioning, and motivation for behavioral change [10].

Taken together, the current literature suggests that physical activity, healthy eating, and psychological well-being interact through multiple interrelated biological pathways—metabolic, hormonal, inflammatory, immunological, and psychoneuroendocrine—that are relevant to the long-term health of breast cancer survivors. A synthesis of evidence on integrated lifestyle interventions addressing these dimensions simultaneously remains needed.

Therefore, the objective of this systematic review was to evaluate the effects of structured diet and physical exercise interventions, including those implemented via digital platforms, on body composition, cardiometabolic biomarkers, functional capacity, and psychological outcomes in women who are breast cancer survivors.

## 2. Materials and Methods

### 2.1. Design

This systematic review was conducted in accordance with the recommendations of the PRISMA (Preferred Reporting Items for Systematic Reviews and Meta-Analyses) statement and the guidelines for systematic reviews [11,12]. The registration number for this systematic review on the PROSPERO platform is CRD420251185869.

### 2.2. Eligibility Criteria

Eligibility criteria were established in accordance with the PICO framework (Population, Intervention, Comparison, Outcomes, and Study Design) to ensure the clinical and methodological relevance of the included studies.

Randomized clinical trials and quasi-experimental studies were considered eligible if they included women with a previous diagnosis of breast cancer across different phases of survivorship (P) and evaluated lifestyle interventions based on diet and physical exercise (I). These interventions could be implemented through in-person sessions, supervised programs, digital platforms, mobile apps, web-based programs, or blended interventions combining different delivery modalities.

The comparison groups (C) consisted of participants who received usual care, general lifestyle recommendations, or minimal interventions without structured supervision. The primary outcomes (O) considered included: body composition (fat mass, lean mass, body weight, or waist circumference), physiological or cardiometabolic parameters (blood glucose, lipid profile, or blood pressure), physical fitness or functional capacity (muscle strength, cardiorespiratory fitness, VO_2_max, or six-minute walk test), quality of life and fatigue, psychological variables (anxiety or depression), adherence to the intervention, and adverse events.

The study population comprised women with a previous clinically confirmed diagnosis of breast cancer at different stages of survivorship. This included individuals who had completed primary treatment (surgery, chemotherapy and/or radiotherapy), those receiving adjuvant endocrine therapy, and long-term survivors in remission. Breast cancer diagnosis was established according to the criteria of the American College of Radiology (ACR) or based on medical records [13].

Studies were excluded if:

(a) The study population did not consist of women who were breast cancer survivors;

(b) The intervention did not include structured components of diet, physical exercise, or both;

(c) The language of publication was not English;

(d) The year of publication was more than ten years prior to the date of the literature search, in order to include recent evidence in the field of lifestyle interventions and cancer rehabilitation;

(e) The study design was observational without intervention, cross-sectional, or qualitative.

### 2.3. Search Strategy

A search strategy was used to identify studies primarily examining the relationship between diet and exercise in breast cancer survivors. Searches were conducted in the following electronic databases: Cochrane, PubMed, Scopus, and Web of Science. The literature search was performed between January 2024 and January 2026, and studies published from January 2016 to January 2026 were considered eligible for inclusion. The search strategy combined MeSH terms and free-text terms related to breast cancer, cancer survival, dietary interventions, and physical exercise, using Boolean operators (AND, OR). The main terms used included: “Breast Neoplasms,” “breast cancer,” “cancer survivors,” “survivors,” “diet,” “nutrition,” “dietary intervention,” “exercise,” “physical activity,” “training,” “exercise therapy,” and “physical fitness.”

In PubMed, the following search strategy was used: (“Breast Neoplasms”[MeSH Terms] OR (“breast cancer”[Title/Abstract] OR “Breast Neoplasms”[Title/Abstract])) AND (“Cancer Survivors”[MeSH Major Topic] OR “Survivors”[MeSH Terms] OR (“Survivors”[Title/Abstract] OR “Cancer Survivors”[Title/Abstract])) AND (“Diet”[MeSH Terms] OR “diet, mediterranean”[MeSH Terms] OR “Diet Therapy”[MeSH Terms] OR “Nutrition Therapy”[MeSH Terms] OR (“nutritional interventions”[Title/Abstract] OR “nutritional therapy”[Title/Abstract]) OR “mediterranean diet”[Title/Abstract]) AND (“Exercise”[MeSH Terms] OR “Exercise Therapy”[MeSH Terms] OR “Physical Fitness”[MeSH Terms] OR “Exercise”[Title/Abstract]).

The search strategy used in Cochrane is also included: (“breast cancer” OR “breast neoplasm*” OR “mammary carcinoma”) AND (“cancer survivor*” OR survivor*) AND (“diet” OR “mediterranean diet” OR “dietary intervention*” OR “nutrition therapy” OR “nutritional intervention*” OR “diet therapy”) AND (“exercise” OR “physical activity” OR “exercise therapy” OR “training program” OR “aerobic training” OR “resistance training”).

The full search strategies are available in Supplementary Materials (Tables S1 and S2). All identified records were imported into a reference management system, and duplicates were removed. The studies were then evaluated through a two-stage screening process: review of titles and abstracts, followed by evaluation of the full text of potentially eligible articles. In addition, a manual search of the reference lists of relevant systematic reviews was conducted to identify additional studies that met the inclusion criteria.

### 2.4. Data Collection

Data collection was conducted in accordance with established research protocols [11,12]. A careful review was performed to confirm that the identified articles addressed the research question and met the inclusion criteria. The search and critical review were conducted independently by two authors (M-M. and N-A.) and compiled using reference management software (Mendeley, Elsevier, The Netherlands). Mendeley Reference Manager (Version 2.91.0) [Software]. Elsevier; 2025. Available at: https://www.mendeley.com (accessed on 27 April 2026).

### 2.5. Data Synthesis

Data extraction was performed and cross-checked by two independent researchers (M-M. and N-A.). In the event of discrepancies, a third researcher (A-M.) was consulted to reach a consensus.

The main results of the included studies were summarized in a table that included the following variables: author (first author, year), country, study design, number of participants, mean age (years), time post-treatment, cancer type, nutritional intervention, duration of nutrition, exercise intervention, duration of exercise, comparison (groups), measured variables, main results, adherence (%), motivational strategies/innovations and limitations.

Due to the clinical and methodological heterogeneity observed among the studies—including differences in the type of intervention (diet alone, exercise alone, or combined interventions), the mode of delivery (in-person, digital, or blended), the duration and intensity of the interventions, and the tools used to measure outcomes—it was not considered methodologically appropriate to conduct a quantitative meta-analysis. Specifically, outcome measures varied substantially across studies: fatigue was assessed using at least three different validated instruments (EORTC QLQ-C30, FACT-B, and the Brief Fatigue Inventory); body composition was quantified by DXA, bioelectrical impedance, or simple anthropometry; and physical activity was captured either objectively (accelerometry) or via self-reported questionnaires, which are prone to social desirability bias. Intervention duration ranged from 8 weeks to 24 months, and study designs included randomized controlled trials, single-arm trials, and pilot feasibility studies with sample sizes from 16 to over 150 participants per group. This combination of variability in populations, protocols, and measurement instruments precluded meaningful statistical pooling and supported the choice of a narrative synthesis approach, in line with current PRISMA-S and Cochrane guidance for reviews with high conceptual heterogeneity.

Therefore, a qualitative narrative synthesis of the findings was conducted, organizing the results according to the main outcome domains assessed in the included studies, such as body composition, cardiometabolic biomarkers, functional capacity, and psychological outcomes.

### 2.6. Methodological Quality

Two researchers (M-M. and N-A.) independently conducted a final analysis to assess the methodological quality of the full-text articles that met the eligibility criteria. Depending on the type of study, different internationally validated and recommended tools were used to assess the risk of bias. The risk of bias in the included studies was assessed based on their methodological design. For randomized clinical trials, the RoB 2.0 tool [14] was used, which analyzes five domains of potential bias. For non-randomized studies, the ROBINS-I tool [15] was applied, which evaluates seven domains related to internal validity. Both tools were developed and validated by the Cochrane Group to ensure methodological quality and transparency in the critical appraisal of the included studies.

In addition, the overall certainty (quality) of the body of evidence for each outcome domain (body composition, metabolic and inflammatory biomarkers, functional capacity and fatigue, quality of life and psychological well-being, and dietary habits) was rated using the Grading of Recommendations Assessment, Development and Evaluation (GRADE) approach [16]. Following the standard GRADE methodology, randomized controlled trials started as high certainty and observational/quasi-experimental designs as low certainty, and were then downgraded (−1 or −2 levels) according to five domains—risk of bias, inconsistency, indirectness, imprecision and publication bias—or upgraded when factors such as a large magnitude of effect, a dose–response gradient or plausible confounders acting against the observed effect were present. The certainty of evidence was finally classified into four levels: high, moderate, low, or very low. Each study was assessed independently by two reviewers, and discrepancies were resolved by consensus. The results of this critical appraisal, including the per-study GRADE and the summary-of-findings table by outcome domain, can be found in the Supplement to this article (Tables S3–S5).

## 3. Results

A total of 1413 records were identified. After removing 455 duplicates, 958 records were screened by title and abstract. Of these, 893 were excluded for not meeting the inclusion criteria. The remaining 65 full-text articles were assessed for eligibility. Of these, 30 were excluded because they were not relevant to the study: studies on diseases other than breast cancer (*n* = 10), books or book chapters (*n* = 2), and studies that did not meet the inclusion criteria (*n* = 18).

The full text of the remaining 35 articles was reviewed. Of these, 20 were excluded for the following reasons: two were systematic reviews, and 18 did not comply with the methodology established in the inclusion criteria defined in this review.

**Figure 1:**
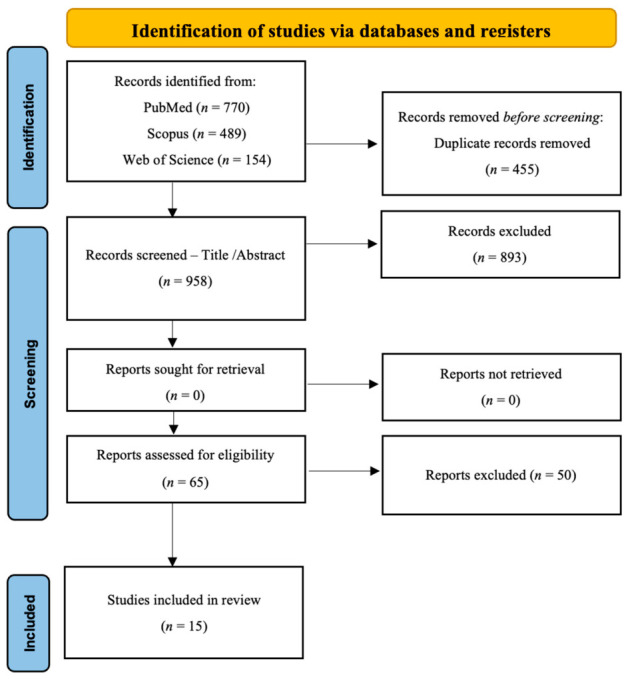
PRISMA diagram: flowchart of the study section’s process [17].

A total of 15 studies were included. The study selection process is summarized in the PRISMA flow diagram (Figure 1).

### 3.1. Summary of the Included Studies

This systematic review included 15 studies (randomized clinical trials, quasi-experimental studies and controlled pilot studies) published between 2016 and 2026 that evaluated nutritional, exercise, or combined lifestyle interventions in women with a history of breast cancer [2,18,19,20,21,22,23,24,25,26,27,28,29,30,31]. The detailed characteristics of the included studies—population, study design, sample size, mean age, follow-up duration, eligibility criteria, post-treatment phase and cancer type—are summarized in Table 1, while the description of the nutritional and exercise components, comparators, variables measured and main results of each trial are presented in Table 2. Most participants were post-treatment (between 0 and 24 months post-treatment), with mean ages ranging from 46 to 60 years, and most studies recruited overweight or obese women. Of the included studies, 11 were randomized controlled trials (RCTs) [2,7,18,21,23,24,25,28,29,30,31], three were single-arm pre–post or pilot studies [19,20,26], and one was a prospective cohort with a modeling approach [22].

Interventions ranged from supervised in-person programs (aerobic exercise, resistance training, yoga or multimodal diet + exercise) [18,19,20,22,23,24,25,26,27,28,30,31] to home-based or web-based strategies [2,21,29], with durations ranging from 12 weeks to 12 months. To facilitate the comparative interpretation of the included evidence, Table 3 presents a methodological synthesis of each study, classifying the intervention type, delivery format (in-person, hybrid or digital), supervision level (supervised, semi-supervised or unsupervised), exercise intensity and dose, duration, main outcomes improved, and overall certainty of the evidence according to the GRADE approach [16]. The most common outcomes evaluated across studies were body composition, metabolic and inflammatory biomarkers, functional capacity and fatigue, quality of life and psychological well-being, and dietary habits, which are analyzed in the following subsections.

**Table 1:** The characteristics of the included studies (population, study design and follow-up).

Author (Year)	Study Design	N	Age	Follow-Up	Population/Inclusion Criteria	Post-Treatment	Cancer Type
Brown J.C., 2021 [18]	RCT	351	Adults	24 weeks	BMI 25–50 kg/m^2^ (overweight/obesity)	≥6 months after primary treatment (hormonal therapy allowed)	Breast cancer (with lymphedema in criteria)
Greenlee H. et al., 2024 [2]	RCT	74	58.4 ± 10.1 years	6 months	≤5 servings/day F&V or <150 min/week MVPA	6–24 months after treatment	Breast cancer stage I–III
Arikawa A., 2018 [23]	RCT	20	Not reported (postmenopausal)	12 weeks	BMI ≥ 27 kg/m^2^	≥3 months after treatment	Breast cancer
Holtdirk F., 2021 [29]	RCT	363	30–70 years	6 months	Breast cancer diagnosis < 5 years	≥1 months post-acute treatment	Breast cancer
Ghavami H., 2017 [28]	RCT	80	48–50 years	6 months	Women with BMI > 25 kg/m^2^, stage I–III, completed treatment 3–18 months prior, on endocrine therapy	3–18 months post-treatment	Breast cancer (stage I–III)
Reis A.D., 2021 [30]	RCT	23	40–75 years	6 months	On hormonal therapy (tamoxifen or aromatase inhibitors), BMI ~29–31	During or after hormonal treatment	Breast cancer
Ruiz-Vozmediano J., 2020 [31]	RCT	63	52 ± 6 years	6 months	Completed treatment > 12 months earlier	>12 months post-treatment	Breast cancer stage IIA–IIB
García-Unciti M., 2023 [27]	RCT	43	55–70 years	12 months	Postmenopausal women, BMI ≤ 35 kg/m^2^	After 1 year of treatment with aromatase inhibitors	Breast cancer (treated with AIs)
Dieli-Conwright C.M., 2018 [25]	RCT	100	53.6 ± 10.4	16 weeks + 3 months follow-up	Sedentary women with BMI ≥ 25 kg/m^2^	≥6 months after primary treatment	Breast cancer (stage 0–III)
Sturgeon K.M., 2018 [21]	RCT	35	46.1 ± 4.0	12 months	BMI 29.9 ± 4.8 kg/m^2^	≥4 months post-treatment + ≥2 years post-prophylactic oophorectomy	Breast cancer, BRCA1/2 carriers
Travier N., 2018 [20]	Pre–post study (phase II, single intervention)	42	18–75 years	12 weeks	BMI ≥ 25 kg/m^2^ (overweight)	0–6 months since end of treatment	Breast cancer (overweight/obese)
Fabian C.J., 2021 [26]	Pilot study/two cohorts	22	60 years	12 weeks	BMI 37 kg/m^2^	Post-treatment (postmenopausal, sedentary)	Breast cancer (postmenopausal obese survivors)
Suzuki H., 2020 [19]	Prospective single-arm pre–post study	32	37–67 years	6 months	Patients on adjuvant endocrine therapy (tamoxifen or AIs)	Mean therapy duration ≈24 months; surgery ≥12 months prior to participation	Breast cancer survivors (various stages; ~half stage I) with weight gain or dyslipidemia
Pistelli M., 2021 [22]	Prospective cohort study	98	Adults	12 months	Majority on hormonal therapy; BMI ≥ 25 kg/m^2^	Post-surgery ± chemo/radiotherapy, under follow-up	Breast cancer (early high-risk, BMI ≥ 25 or metabolic syndrome)
Buckland G., 2019 [24]	RCT	37	55.1 ± 8.3 years	12 weeks	30.5 ± 3.9 kg/m^2^	3 months post-chemotherapy/radiotherapy	Breast cancer (stage I–IIIA, overweight/obese)

Abbreviations: AIs, aromatase inhibitors; BMI, body mass index; BRCA1/2, breast cancer gene 1/2; F&V, fruit and vegetables; MVPA, moderate-to-vigorous physical activity; RCT, randomized controlled trial.

**Table 2:** The intervention components and main results of the included studies.

Author (Year)	Nutritional Intervention	Exercise Intervention	Comparator	Variables Measured	Main Results
Brown J.C., 2021 [18]	Structured hypocaloric program (diet)—counseling and lifestyle sessions	Supervised exercise + home-based program (resistance + aerobic)	Control (usual care)	Weight, DXA (fat/lean mass), VAT, BMI, BMD, lymphedema	Diet alone and diet + exercise: clinically meaningful weight loss (5.4 kg and −6.7 kg at 52 weeks). Exercise alone did not change weight; reduction in fat and VAT in diet arms. No effect on BMD.
Greenlee H. et al., 2024 [2]	Online Mediterranean-style dietary program with Zoom sessions and digital materials	Multicomponent training (aerobic + strength) via Zoom; Fitbit monitoring	Low-dose virtual control	Weight, BMI, MVPA, diet, QoL, feasibility	High-dose group increased F&V (+1.5 servings/day) and reduced calories and fat; no changes in MVPA, BMI.
Arikawa A., 2018 [23]	Provided meals (~1000 kcal/day deficit; 55% CHO, 15% protein, 30% fat) vs. counseling	Supervised aerobic + strength training	Weight management counseling	Weight, BMI, DXA, fitness (METs), insulin, glucose, IGF-1, CRP, IL-6, vitamin D, QoL, sleep	Greater weight loss (~11%) and improved fitness and biomarkers in intervention; reduced sleep quality and social scores.
Holtdirk F., 2021 [29]	Online content on diet, exercise, sleep, stress	Behavioral advice on PA (no structured training program)	Usual care/wait-list	QoL, diet, PA, anxiety, depression, stress, sleep	Improvement in QoL (d = 0.27) and dietary habits (d = 0.36); no significant effect on PA.
Ghavami H., 2017 [28]	Energy restriction program + nutritional education	Supervised aerobic + resistance training (≥3 sessions/week)	Usual care/control	EORTC QLQ-C30 (QoL), anthropometry, PA	Intervention significantly improved global QoL (*p* < 0.001) and other subscales; clinically relevant improvements in well-being.
Reis A.D., 2021 [30]	Monthly personalized dietary counseling	Supervised aerobic exercise (150 min/week; HR-controlled)	FF vs. HB	Diet (3-day record), PA (Baecke), BIA, lipids, cardiopulmonary fitness	Both groups showed reduced lipids, increased PA and cardiorespiratory fitness; no changes in BIA.
Ruiz-Vozmediano J., 2020 [31]	Multidisciplinary program: Mediterranean diet workshops, adherence assessment, dietary counseling	Exercise program (21 sessions) + 8 mindfulness sessions	Control (general recommendations)	Weight, BMI, Mediterranean diet adherence, QoL (EORTC QLQ-C30), lipids, tumor markers	IG showed improvements in physical and role functioning, increased adherence to MD (*p* < 0.001), ↓ BMI, improved lipid profile.
García-Unciti M., 2023 [27]	Dietary advice and habit assessment (MD)	Combined training: 1 h impact-aerobic + 1 h strength (2×/week)	CG (usual care with Ca/D supplementation)	MRI (VAT/subcutaneous fat), anthropometry, dietary questionnaires (MD adherence)	IG showed significant reductions in VAT, subcutaneous and total fat; improved body composition; moderate adherence to Mediterranean diet; micronutrient deficiencies identified.
Dieli-Conwright C.M., 2018 [25]	No intensive dietary intervention (only 3-day dietary records at different time points)	Supervised combined aerobic + resistance training (3 sessions/week, 65–85% HRmax)	Usual care (standard advice)	Metabolic syndrome z-score, DXA, strength, estimated VO_2_, biomarkers (insulin, IGF-1, leptin, adiponectin), anthropometry	Significant improvements in metabolic syndrome z-score, reduction in fat mass, improvements in adipokines and body composition; effects maintained at 3 months.
Sturgeon K.M., 2018 [21]	Precision Nutrition web-based program: biweekly habits + daily educational materials	Home-based mixed exercise: 160 min/week (3 days strength, 2 days aerobic intervals, 1 active recovery)	Wait-list control group (usual habits)	DXA (SAT, VAT, TAT, lean mass), insulin, IL-1β, IL-6, IL-8, TNF-α	↓ insulin and SAT; ↓ TNF-α within group; no changes in IL-6/IL-1β/IL-8; correlation between insulin and SAT changes.
Travier N., 2018 [20]	Healthy diet education and sessions (nutritional counseling)	Exercise sessions + improvement in CRF (3×/week)	No CG (pre–post)	Anthropometry, VO_2_peak, insulin, HOMA-IR, leptin/adiponectin, lipids	Significant improvements in metabolic biomarkers and insulin resistance; mean weight loss ~5.6 kg in prior intervention; correlation between BMI reduction and improvements in leptin/CRF.
Fabian C.J., 2021 [26]	Caloric restriction + dietary control	Rapid escalation to high MVPA (≥200 min/week) with trainers (2×/week), monitoring (Garmin, MyFitnessPal)	No randomized control	DXA (VAT), adipokines, insulin, VO_2_peak, weight, MVPA by accelerometer	Median VAT −19%; total mass −8%; adiponectin:leptin ratio improved from 0.77 (below normal) to 1.08 (normal); maintenance of high MVPA after trainer withdrawal (9/11 continued).
Suzuki H., 2020 [19]	Brief nutritional education (15 min) by dietitian; food logging and self-monitoring	3 group aerobic exercise sessions (45 min each: 30 min jogging/dance + 15 min strength/yoga), supervised weekly (3 weeks); instructional home exercise video	No CG	Body weight, BMI, arm circumference, triceps skinfold, AST/ALT, CHO, TG, K6 (psychological distress), CFS, SES	At 1 month: ↓ weight (*p* < 0.01), ↓ BMI (*p* < 0.01), ↓ TG (*p* < 0.05), ↓ CHO (*p* < 0.01), ↓ distress (K6 *p* < 0.05) and ↓ CFS *p* < 0.01). At 3 and 6 months: continued reductions in weight, BMI and triceps skinfold; some improvements in CHO/CFS and SES at 6 months.
Pistelli M., 2021 [22]	MD, caloric restriction, nutritional education	Moderate PA: ≥150 min/week + resistance training 2×/week; multidisciplinary supervision	No formal CG; cohort follow-up	BMI, waist circumference, glucose, insulin, testosterone, lipid profile, HADS, joint pain	↓ BMI, waist, glucose, insulin, testosterone; ↑ psychosocial well-being; ↓ HADS, ↓ joint pain.
Buckland G., 2019 [24]	Weekly 1 h group sessions with dietitian; hypocaloric MD; focus on reducing saturated fat and increasing fish/vegetables	75 min, 2×/week, aerobic (cycling) + supervised strength (bands, balls, mats)	No CG	Dietary intake (3 × 24 h), BMI, BIA, plasma carotenoids, erythrocyte membrane fatty acids	↓ saturated fat (−1.4%), ↑ MUFA (+1.7%), ↑ omega-3 (+13%), improved *n*-6/*n*-3 ratio; increased dietary and plasma carotenoids.

Abbreviations: IG: intervention group; CG: control group; EORTC: The European Organization for Research and Treatment of Cancer; HADS: Hospital Anxiety and Depression Scale; F&V = fruit and vegetables; BMI: body mass index; DXA: dual-energy X-ray absorptiometry; BMD: bone mineral density; VAT: visceral adipose tissue; AIs: aromatase inhibitors; ALT: alanine aminotransferase; AST: aspartate aminotransferase; CHO: total cholesterol; CFS: Cancer Fatigue Scale; SES: Self-Efficacy Scale for Cancer Patients; K6: Kessler 6-item Psychological Distress Scale; MD: Mediterranean diet; MS: metabolic syndrome; CRF: cardiorespiratory fitness; HOMA-IR: insulin resistance; VO_2_peak: peak oxygen uptake; PA: physical activity; MVPA: moderate-to-vigorous physical activity; ACSM/ACS: American College of Sports Medicine/American Cancer Society; IGF-1: insulin-like growth factor-1; MUFA: monounsaturated fatty acids; SAT: subcutaneous adipose tissue; TAT: total adipose tissue; HRmax: maximum heart rate; FF: face-to-face training group; HB: home-based training; ↓: Decrease in value compared to the initial value; ↑: Increase in value compared to the initial value.

**Table 3:** Methodological synthesis of intervention features and overall certainty of evidence (GRADE) for each included study.

Study	Intervention Type	Delivery Format	Supervision Level	Exercise Intensity	Duration	Main Outcomes Improved	GRADE
Brown J.C., 2021 [18]	Diet ± Exercise (4 arms)	FF + home-based	Supervised + semi-supervised	Aerobic + resistance, ACSM	24–52 weeks	↓ Weight (−5.4 to −6.7 kg); ↓ total fat; ↓ VAT (in diet arms)	MODERATE
Greenlee H. et al., 2024 [2]	Digital multimodal (diet + exercise)	Online (Zoom + Fitbit)	Semi-supervised (virtual)	Aerobic + resistance, moderate	6 months	↑ Fruit/vegetable intake (+1.5 servings/day); ↓ kcal and fat; no changes in MVPA, BMI	MODERATE
Arikawa A., 2018 [23]	Diet + Exercise (comidas + ejercicio)	FF	Supervised	Aerobic + resistance, moderate–vigorous	12 weeks	↓ Peso (~11%); ↑ fitness; ↓ insulin, IGF-1, CRP; ↑ vitamin D	LOW
Holtdirk F., 2021 [29]	Digital multimodal (online CBT)	Online (plataforma Optimune)	Unsupervised	Behavioral PA counseling (no structured program)	6 months	↑ QoL (d = 0.27); ↑ dietary habits (d = 0.36); no effect on PA	LOW
Ghavami H., 2017 [28]	Diet + Exercise	FF	Supervised	Aerobic + resistance ≥3 sessions/week	24 weeks	↑ QoL global (EORTC QLQ-C30, *p* < 0.001); ↑ functional subscales	VERY LOW
Reis A.D., 2021 [30]	Diet + Exercise (FF vs. HB)	FF vs.—HB	Supervised vs. semi-supervised	Aerobic 150 min/week (HR control)	24 weeks	↓ Lipids; ↑ PA and cardiorespiratory fitness in both groups	LOW
Ruiz-Vozmediano J., 2020 [31]	Diet + Exercise + Mindfulness	FF	Supervised	Structured program (21 sessions) + 8 mindfulness sessions	6 months	↑ Physical/role/social functioning; ↑ MD diet adherence; ↓ IMC; ↑ lipid profile	LOW–MODERATE
García-Unciti M., 2023 [27]	Exercise (with dietary assessment)	FF	Supervised	Impact-aerobic (1 h) + resistance (1 h), 2×/week	12 months	↓ VAT, ↓ SAT, ↓ total fat and waist circumference	LOW
Dieli-Conwright C.M., 2018 [25]	Exercise (combined)	FF (one-to-one)	Supervised	Aerobic + resistance, 3×/week, 65–85% HRmax	16 weeks + 3-month follow-up	↓ MetS z-score; ↓ sarcopenic obesity; ↓ insulin, IGF-1, leptin; ↑ adiponectina	MODERATE–HIGH
Sturgeon K.M., 2018 [21]	Digital multimodal (Precision Nutrition)	Online (web)	Unsupervised (with virtual coach)	160 min/week (3 resistance, 2 aerobic intervals, 1 recovery)	12 months	↓ SAT y total fat; ↓ insulin; ↓ TNF-α within-group; no changes in IL-6/8, VAT	VERY LOW
Travier N., 2018 [20]	Diet + Exercise	FF	Supervised	Exercise 3×/week + CRF improvement	12 weeks	↑ Metabolic profile, ↓ HOMA-IR, ↓ leptin; ↓ weight (~5.6 kg, prior study)	VERY LOW
Fabian C.J., 2021 [26]	Diet + Exercise (restriction + MVPA)	FF + digital (Garmin/MyFitnessPal)	Semi-supervised (trainer 2×/week→withdrawal)	MVPA ≥ 200 min/week (moderate–vigorous)	12–24 weeks	↓ VAT (−19% at 12 wk, −29% at 24 wk); ↓ masa (−8 a −12%); ↑ adiponectin:leptin ratio	VERY LOW
Suzuki H., 2020 [19]	Diet + Exercise + Group coaching	FF + home video	Semi-supervised	3 group aerobic sessions (45 min: jogging/dance + resistance/yoga)	6 months	↓ Peso, IMC, TG, colesterol; ↓ distress (K6); ↓ fatiga (CFS); ↑ autoeficacia (SES)	VERY LOW
Pistelli M., 2021 [22]	Diet + Exercise (multidisciplinary)	FF (hospital)	Supervised	≥150 min/week moderate + resistance 2×/week	12 months	↓ BMI, waist circumference, glucose, insulin, testosterone; ↑ well-being; ↓ HADS; ↓ dolor articular	VERY LOW
Buckland G., 2019 [24]	Diet + Exercise	FF (hospital group)	Supervised	Aerobic (cycling) + resistance, 75 min, 2×/week	12 weeks	↑ Carotenoids; ↓ SFA; ↑ MUFA y *n*-3 PUFA; ↓ *n*-6/*n*-3 ratio	VERY LOW

Abbreviations: ACSM: American College of Sports Medicine; BMI: body mass index; CBT: cognitive behavioral therapy; CRF: cardiorespiratory fitness; EORTC QLQ-C30: European Organization for Research and Treatment of Cancer Quality of Life Questionnaire; FF: face-to-face; HADS: Hospital Anxiety and Depression Scale; HB: home-based; HOMA-IR: Homeostatic Model Assessment of Insulin Resistance; HRmax: maximum heart rate; MD: Mediterranean diet; MUFA: monounsaturated fatty acids; MVPA: moderate-to-vigorous physical activity; *n*-3 PUFA: Omega-3 polyunsaturated fatty acids; PA: physical activity; QoL: quality of life; SAT: subcutaneous adipose tissue; SFA: saturated fatty acids; Smet: metabolic syndrome; VAT: visceral adipose tissue; ↓: Decrease in value compared to the initial value; ↑: Increase in value compared to the initial value.

### 3.2. Effect on Body Composition Parameters

Taking together, the included trials show that lifestyle interventions combining calorie restriction with structured exercise (aerobic and/or strength training) result in clinically significant reductions in body weight and adiposity. In the RCTs, weight loss ranged from 5% to 8% of baseline weight, accompanied by significant decreases in total and visceral fat mass compared with control groups [18,28,31]. When analyzed by intervention type, multimodal programs (diet + supervised exercise) showed the most reproducible patterns, with reported reductions of up to 7.7% in body weight and ~10% in body fat [20]. Furthermore, several studies described associations between higher levels of physical activity, lower adipose tissue, and improvements in the adipokine profile [18,26,27].

In specific subgroups, such as postmenopausal women undergoing treatment with aromatase inhibitors, combined programs of aerobic and high-impact exercise significantly reduced subcutaneous and visceral adipose tissue (VAT), as well as waist circumference [19]. Similarly, interventions targeting women with high metabolic risk following primary surgery showed improvements in waist circumference along with favorable changes in metabolic parameters [22].

In contrast, digital or eHealth interventions showed more reliable effects on dietary behaviors than on objective anthropometric outcomes. Improvements in energy intake and diet quality were observed, whereas changes in body weight, BMI, or objectively measured physical activity were often not statistically significant between groups [2,29].

Regarding intervention components, diet-only approaches were associated with modest reductions in body weight but also with decreases in lean body mass (0.8–1.2 kg over 12 months) [18], whereas combined interventions tended to better preserve body composition. Despite these trends, the magnitude of effects varied considerably across studies depending on intervention intensity, supervision, and duration.

Across the included studies, lifestyle interventions produced significant changes in body composition in 12 of the 14 trials that assessed this domain (86%). The magnitude and direction of the effect, however, varied substantially according to the intervention subgroup.

Multimodal supervised (diet + exercise): The largest and most pronounced reductions in body weight, total fat mass and VAT were observed in multimodal supervised programs combining structured diet and exercise. Weight loss ranged from 5% to 8% of baseline (with peaks of 11% and 5.4–6.7 kg at 52 weeks) [18,23] and was accompanied by significant decreases in VAT (up to −19% at 12 weeks and −29% at 24 weeks) [26], subcutaneous adipose tissue and waist circumference [18,19,20,22,23,24,26,31].

Exercise alone (combined aerobic + resistance): Programs based on supervised combined aerobic and resistance training without intensive dietary intervention also achieved clinically relevant reductions in VAT, SAT and total fat mass while preserving or improving lean mass [25,27]. Dieli-Conwright et al. [25] additionally demonstrated significant improvements in sarcopenic obesity, indicating that exercise can modify body composition independently of caloric restriction.

Diet alone: In the only included trial with a diet-only arm, weight loss was achieved but was accompanied by a concomitant decrease in lean body mass (0.8–1.2 kg over 12 months), underscoring the risk of muscle mass loss when caloric restriction is not paired with resistance training [18].

Digital/eHealth: Digital interventions showed heterogeneous and generally smaller effects on objective body composition outcomes. Greenlee et al. [2] and Holtdirk et al. [29] reported no significant changes in BMI or body weight, whereas Sturgeon et al. [21] did show significant reductions in subcutaneous and total adipose tissue without changes in visceral fat. Across digital trials, improvements in dietary behaviors were more reliable than changes in objective anthropometric measures. Detailed magnitudes of effect, consistency and GRADE certainty by intervention type are summarized in Table 4.

### 3.3. Effects on Metabolic Biomarkers and Adipokines

Of the 11 studies that measured metabolic biomarkers or adipokines, nine reported significant improvements. The magnitude of these changes depended primarily on the intensity of the intervention and on the level of supervision.

Multimodal supervised (diet + exercise): Multimodal supervised programs produced the broadest and most comprehensive metabolic profile. In 12–16-week interventions, fasting insulin decreased by 15% (*p* = 0.008) and HOMA-IR by 13.5% (*p* = 0.007), with parallel reductions in triglycerides (−20.2%), total cholesterol (−9.4%) and LDL-C (−4.3%) [20,23,24,30,31]. In longer-term programs (≥6 months), Pistelli et al. [22] reported sustained reductions in serum insulin, testosterone and lipid profile from 6 to 12 months (*p* < 0.0001), and Suzuki et al. [19] observed early reductions in triglycerides and total cholesterol within the first month. Others, additionally, showed normalization of the adiponectin: leptin ratio (from 0.77 to 1.08) [26].

Exercise alone (supervised combined): Dieli-Conwright et al. [25] provide the strongest single-trial evidence for the metabolic impact of structured exercise without dietary restriction: 16 weeks of supervised combined training significantly improved fasting insulin, IGF-1, leptin and adiponectin, and reduced the overall metabolic syndrome z-score (all *p* < 0.001 vs. control), with effects maintained at 3-month follow-up.

Digital/eHealth: Digital interventions produced smaller and more variable metabolic changes. Sturgeon et al. [21] observed a moderate correlation between reductions in insulin and SAT (r = 0.33) but no significant changes in IL-1β, IL-6 or IL-8; only TNF-α decreased within groups. Detailed effects and GRADE certainty by intervention type are summarized in Table 5.

### 3.4. Effects on Functional Capacity and Fatigue

Improvements in functional capacity and reductions in cancer-related fatigue were observed across most included studies [2,19,20,22,23,28,29,31], but their magnitude was strongly associated with the type and intensity of the intervention.

Multimodal supervised (diet + exercise): Programs combining diet and supervised exercise produced the largest improvements in objective functional measures. Pistelli et al. [22] reported an increase in 6 min walk test completion from 17.3% to 73.5% over 12 months; Ghavami et al. [28] and Ruiz-Vozmediano et al. [31] described substantial increases in EORTC QLQ-C30 functional scales (Δ +25–34 points); and Dieli-Conwright et al. [25] documented significant gains in muscle strength and estimated VO_2_peak. Fatigue scores improved in parallel, with absolute reductions of up to −39.44 points in the EORTC QLQ-C30 [28] and sustained decreases in the Cancer Fatigue Scale [19] and HADS [22].

Exercise alone or with mind–body component: Exercise-based programs [25,27] and those that incorporated a mind–body component (mindfulness [31]; group yoga/dance [19]) showed moderate to large improvements in cardiorespiratory fitness, muscle strength and fatigue, supporting the value of mind–body adjuncts in the management of cancer-related fatigue.

Digital/eHealth: Digital interventions produced smaller effects. Greenlee et al. [2] found no significant difference in PROMIS physical function between high- and low-dose groups (adjusted difference −0.56; 95% CI −1.27 to 0.15) and no significant change in objectively measured MVPA. Holtdirk et al. [29] reported a small but significant improvement in quality of life (d = 0.27; 95% CI 0.07–0.48) as a primary endpoint; the effect on physical activity (d = 0.30; 95% CI 0.10–0.51) did not reach significance after Bonferroni adjustment (α = 0.0167 for three primary endpoints), and cancer-related fatigue, assessed as a secondary outcome, showed only a modest between-group effect (d = 0.23; 95% CI 0.02–0.44).

While some interventions produced very pronounced reductions in fatigue scores, the evidence suggests a heterogeneous response rather than a uniformly large effect. Differences in intervention intensity, exercise supervision, duration, and measurement instruments may partially explain the variability observed across studies. Consequently, isolated large improvements should not be interpreted as representative of all lifestyle interventions in breast cancer survivors. Magnitudes of effect by intervention type are summarized in Table 6.

### 3.5. Effects on Quality of Life and Psychological Well-Being

Of the eight studies that formally assessed quality of life or psychological well-being using validated scales, 75% reported significant improvements. The magnitude varied markedly according to the intervention subgroup.

Multimodal supervised (diet + exercise): Long-term multimodal programs generated the largest improvements in both global quality of life and specific functional domains. Ghavami et al. [28] reported increases in general health status from 57.5 ± 15.2 to 91.3 ± 8.0 points (intervention vs. virtually unchanged in the control group; *p* < 0.001), with parallel reductions in fatigue (from 42.5 ± 23.5 to 3.1 ± 5.6), pain (from 40.4 ± 24.7 to 4.2 ± 7.3) and nausea/vomiting (from 14.6 ± 19.7 to 1.7 ± 5.0). Ruiz-Vozmediano et al. [31] observed sustained improvements in physical (Δ +9.6 pts) and role functioning (Δ +9.2 pts) at 6 months. Pistelli et al. [22] described significant reductions in HADS scores, while Suzuki et al. [19] demonstrated improvements in K6 distress and SESs.

Digital/eHealth: Digital interventions produced smaller effects (d = 0.27), with improvements limited in quality of life (d = 0.27; 95% CI: 0.07–0.48) along with reductions in insomnia, fatigue, and depressive symptoms, suggesting that digital effects tend to be of small-to-moderate magnitude and dependent on program content [29]. Greenlee et al. [2] found no significant changes in physical functioning (−0.56 points; *p* = 0.12) or anxiety (+2.53 points; *p* = 0.10) after 6 months of eHealth intervention. Detailed effects by intervention type are summarized in Table 7.

### 3.6. Effects on Eating Habits

All trials reporting on dietary outcomes showed improvements in at least one component of dietary behavior or diet quality, although the magnitude and biological validity of these changes were highest in multimodal supervised programs.

Multimodal supervised (diet + exercise): The clearest and most biologically validated dietary changes were observed in programs that combined nutritional counseling with supervised exercise. Buckland et al. [24] documented a +15.1% increase in dietary carotenoids and a +5–9% increase in B-vitamin intake, accompanied by objective shifts in erythrocyte fatty acid composition (−1.4% saturated fatty acids, +1.7% MUFA, +13% *n*-3 PUFA, ↓ *n*-6/*n*-3 ratio). Reis et al. [30] reported reductions in total energy intake and polyunsaturated fat and increases in physical activity and cardiorespiratory fitness in both face-to-face and home-based arms; the home-based arm additionally increased folic acid intake (*p* < 0.05), whereas the face-to-face arm achieved additional reductions in saturated fat and sodium. Ruiz-Vozmediano et al. [31] described a +1.57-point increase in Mediterranean diet adherence (*p* < 0.001). Energy intake reductions across multimodal trials ranged from ≈170 kcal/day to ≈20% of total intake (equivalent to a 15–20% average decrease), with concomitant reductions in total and saturated fat of 10–31% [20,22,23].

Diet alone or with light exercise advice: García-Unciti et al. [27] found that, in the absence of intensive nutritional counseling, most participants maintained only moderate adherence to the Mediterranean diet (77% moderate; only 19% high adherence), and persistent deficits in calcium, zinc, folate and vitamins D, A and E were identified. The diet-only arm produced modest changes in body weight without substantial improvements in diet quality [18].

Digital/eHealth: Digital programs modified specific dietary behaviors, although their results were more variable. Greenlee et al. [2] demonstrated a high-dose group increase in fruit and vegetable intake of +1.5 servings/day plus reductions in calories and fat; Holtdirk et al. [29] reported a moderate effect on global dietary habits (d = 0.36); and Sturgeon et al. [21] documented behavioral improvements without including objective biomarkers of diet quality. Detailed effects by intervention type are summarized in Table 8.

### 3.7. Inflammatory and Immune Markers

Five included studies measured inflammatory or immune-related biomarkers, with results that varied according to the modality and the dose of the intervention.

Multimodal supervised (diet + exercise): Arikawa et al. [23] reported substantial reductions in leptin (47 to 19 ng/mL at week 12; rebound to 26.2 ng/mL at week 18 during follow-up), F2-isoprostanes (71.5 to 50 ng/mL) and CRP in the caloric-restriction + exercise arm, whereas IL-6 and adiponectin did not show significant changes; IGF-1 paradoxically increased with weight loss, consistent with the inverse association between BMI and IGF-1 reported by the authors. Fabian et al. [26] demonstrated normalization of the adiponectin: leptin ratio (from 0.77, below normal, to 1.08, within normal range). These findings suggest an anti-inflammatory trend in supervised multimodal interventions, although the number of trials directly assessing inflammatory markers remains limited.

Exercise alone (supervised combined): Dieli-Conwright et al. [25] provides the broadest and strongest evidence for a direct anti-inflammatory effect of supervised exercise without dietary restriction, with significant decreases in IL-6, IL-8, TNF-α and high-sensitivity CRP (all *p* < 0.001 vs. control), accompanied by reductions in insulin, IGF-1 and leptin and increases in adiponectin, with effects maintained at 3-month follow-up.

Digital/eHealth: Sturgeon et al. [21] showed reductions in insulin and subcutaneous adipose tissue but no significant change in IL-1β, IL-6 or IL-8; only TNF-α decreased within groups. This pattern suggests that the magnitude of body composition change achievable through digital platforms may be insufficient to substantially modify systemic inflammation. Detailed effects by intervention type are summarized in Table 9.

## 4. Discussion

### 4.1. Overview of Findings

The evidence synthesized in this review suggests that lifestyle interventions integrating structured nutritional counseling and planned physical exercise are associated with favorable changes in body composition, cardiometabolic profile and several dimensions of well-being in breast cancer survivors [18,20,25,31]. Larger effects were observed when guided energy restriction was paired with supervised aerobic plus resistance training delivered in person, consistent with the ACSM Roundtable consensus by Campbell et al. [32] and with the 2022 American Cancer Society guideline by Rock et al. [33]. These conclusions must, however, be interpreted with caution. Of the 15 studies included, only 11 were randomized controlled trials, while four were single-arm pre–post, pilot or prospective cohort designs without a concurrent comparator. Risk of bias (RoB 2/ROBINS-I) [14,15] and overall certainty of evidence (GRADE) [16] ranged from moderate–high in well-conducted RCTs [25] to very low in single-arm trials [20,22,24,26]. The methodological heterogeneity across interventions and study designs precludes firm conclusions on the superiority of multimodal strategies over unimodal or less structured approaches. Any inference of causal effectiveness should therefore be tempered by the variable methodological quality and the predominance of intermediate biomarker outcomes.

### 4.2. Effect on Body Composition Parameters

Weight loss outcomes—approximately 6–7% at 52 weeks with combined diet and exercise—[18] align with those described in large behavioral trials in survivors with overweight or obesity such as the ENERGY [34], in which clinically relevant weight loss was sustained at 12 months. These data support the value of integrating guided energy restriction with structured exercise and ongoing behavioral follow-up.

Beyond absolute weight loss, the reduction in visceral adipose tissue may represent the most clinically meaningful body composition change, given its strong association with cardiometabolic and adipokine profiles [35]. Combined aerobic plus resistance training reliably improves both body composition and cardiometabolic risk in overweight or obese survivors. For patient education, achieving less visceral fat and more lean mass carries greater prognostic relevance than simply preventing weight regain.

Long-term trials such as Brown et al. have shown a concomitant loss of lean body mass when energy restriction is not paired with adequate strength training or protein-sparing nutritional strategies [18]. This finding reinforces the need to prioritize the quality of body composition change, targeting visceral fat reduction without compromising muscle mass, particularly in survivors on endocrine therapy or at risk of sarcopenia. Optimal exercise intensity, progressive resistance training, and lean mass preservation are therefore key elements for maximizing long-term metabolic and functional benefit, consistent with current exercise oncology recommendations [32,36].

### 4.3. Effects on Metabolic Biomarkers and Adipokines

The most reliable improvements in cardiometabolic biomarkers occurred when interventions achieved a meaningful reduction in visceral adiposity and high program adherence. Fabian et al. [26] (single-arm; very low certainty) reported that visceral fat loss was accompanied by improvements in the adipokine profile and metabolic markers, including normalization of the adiponectin-to-leptin ratio. These findings, while hypothesis-generating, suggest that the quality of body composition change—rather than absolute weight loss—may be a stronger determinant of metabolic risk modulation.

Scott et al. [3] reported decreases in leptin, total cholesterol and diastolic blood pressure with parallel improvements in quality of life, even with modest weight change, suggesting that metabolic benefits do not depend exclusively on the magnitude of weight loss. Similarly, the Yale Exercise and Survivorship (YES) trial by Irwin et al. [5] showed that 150 min per week of aerobic exercise over six months reduced IGF-axis markers (IGF-I and IGFBP-3) and produced a between-group difference in insulin (*p* = 0.089), supporting plausible mechanisms by which physical activity could influence metabolic prognosis. Ligibel et al. [4] reported reductions in insulin after combined aerobic and resistance training, reinforcing the plausibility of multimodal interventions acting on relevant metabolic pathways.

Inflammatory and immune marker findings are discussed in Section 4.6. The recent umbrella review by Zhao et al. [37] confirms that exercise improves several adjuvant-treatment-related symptoms in breast cancer patients and supports a dose–response relationship with objective MVPA measurement.

### 4.4. Effects on Functional Capacity, Fatigue, Quality of Life and Well-Being

The included multimodal interventions reported improvements of varying magnitude in functional capacity, cancer-related fatigue, quality of life and psychological well-being. As a hypothetical bio-behavioral framework drawn from outside our review, Rogers et al. [9] proposed that exercise effects on fatigue may be mediated by activity volume and sleep quality, and modulated by inflammatory markers including IL-6 and IL-10.

Earlier digital interventions, such as the web-based self-care program, achieved higher proportions of participants meeting physical activity recommendations (≥150 min/week) [10] with parallel improvements in diet quality and health-related quality-of-life domains including fatigue. More recent trials, indicate that modifying dietary intake may be more feasible than increasing objectively measured MVPA [2]. The gap between self-reported and accelerometer-measured activity may account for part of the between-study heterogeneity and reinforces the need to incorporate objective measures in future research [36,37]. Digital platforms appear effective for improving fruit and vegetable intake but less effective than in-person supervised sessions for increasing objectively measured MVPA—a finding with direct implications for the design of hybrid programs that prioritize exercise adherence.

Cross-study comparisons indicate that intervention intensity and structure account for a substantial share of the heterogeneity observed in clinical and metabolic outcomes. Interventions with regular follow-up and defined goals, such as those described by Scott et al. [3], produced reliable changes in cardiometabolic parameters and quality of life. Interventions that progressively escalated MVPA with behavioral support—as in Fabian et al. [26] and similar designs such as Ghavami et al. [28]—reached activity levels ≥200 min/week and showed dose–response relationships with visceral fat reduction and adipokine improvements. By contrast, digital interventions offer scalability advantages but may require additional components to elicit meaningful increases in MVPA and remain more susceptible to data loss and self-reporting bias [26].

### 4.5. Effects on Eating Habits

Dietary changes reported in the included trials, particularly those described by Buckland et al. [24], show that quantifiable improvements in diet quality are achievable in breast cancer survivors, including changes in lipid profile and objective nutritional biomarkers. These findings align with prior evidence on the cardiometabolic benefits of Mediterranean dietary patterns (WHEL) [38]. However, large-scale trials examining diet and cancer recurrence have yielded mixed results: the WHEL trial [38] showed no significant reduction in recurrence with a diet high in fruit, vegetables and fiber, while the WINS trial [39] reported signals of benefit during the active intervention period with dietary fat reduction. These data point to a favorable metabolic impact of dietary patterns without firm evidence of a direct reduction in recurrence.

We note, as a brief hypothetical background, that experimental work by some researchers [40,41,42] suggest that certain dietary compounds may modulate enzymes involved in estrogen metabolism (e.g., sulfotransferases, SULT). These mechanisms were not assessed by any of the studies included in this review and are therefore not part of the synthesized evidence. The same caveat applies to omega-3 fatty acid supplementation, which was not tested as an independent intervention in the included trials; both topics are mentioned only as hypothesis-generating areas for future research.

### 4.6. Inflammatory and Immune Markers

The most informative data on insulin resistance and inflammation in the included trials came from three contrasting interventions. Pistelli et al. [22] (low-to-moderate certainty), in a 12-month multidisciplinary cohort, reported progressive reductions in hyperinsulinemia and hyperglycemia, with concurrent decreases in serum insulin and testosterone. Sturgeon et al. [21] (very low certainty), in a 12-month web-based diet and home-based exercise program, observed significant reductions in fasting insulin and subcutaneous adipose tissue. TNF-α decreased significantly within the intervention group, whereas IL-1β, IL-6 and IL-8 showed no significant changes, suggesting that the modest body composition change achievable through digital platforms may be insufficient to modulate systemic inflammation comprehensively. Hagstrom et al. [6] provided a mechanistic complement: a thrice-weekly resistance training program produced cellular-level changes (elevated TNF-α expression in NK/NKT cells) without changes in circulating CRP, IL-6 or IL-10, suggesting anti-inflammatory effects may emerge first at the immune cellular level.

By contrast, Dieli-Conwright et al. [25]—the only included trial of supervised exercise without concurrent dietary intervention—reported the broadest panel of effects. These included significant improvements in metabolic syndrome z-score and sarcopenic obesity, together with reductions in insulin, HOMA-IR, leptin, IGF-1, CRP, IL-6, IL-8 and TNF-α and increases in adiponectin (all *p* < 0.001 vs. control), maintained at 3-month follow-up. Although this pattern is consistent with a direct anti-inflammatory and metabolic effect of supervised exercise independent of caloric restriction, it derives from a single high-quality RCT in a relatively small and ethnically specific sample and therefore requires replication before being considered generalizable.

Regarding fatty acid profile, Buckland et al. [24] showed that a combined hypocaloric Mediterranean diet and exercise program produced significant changes in erythrocyte membrane fatty acid composition—reductions in saturated and *n*-6 PUFA and increases in *n*-3 PUFA and MUFA—coherent with the self-reported dietary changes. This finding identifies the dietary component as the main driver of the fatty acid profile, with potential prognostic implications.

A direct comparison of diet alone, exercise alone and the combination within the same population cannot be drawn from the included studies, as none used such a three-arm design. Nevertheless, the contrast between Dieli-Conwright et al. [25] (exercise without dietary intervention, broad metabolic and inflammatory improvements) and the digital interventions in which dietary change was the main lever [21] is biologically consistent with an additive effect: caloric restriction reduces adiposity and improves insulin sensitivity through negative energy balance, whereas exercise acts independently through skeletal muscle glucose uptake, lean mass preservation and modulation of pro-inflammatory adipokines. For clinical practice, interventions that prioritize only dietary modification—particularly those delivered digitally without supervised exercise—may be insufficient to produce relevant anti-inflammatory benefits in breast cancer survivors, highlighting the role of supervised exercise as an indispensable component of multimodal programs [32,36].

### 4.7. Clinical Implications

From a clinical perspective, these findings reinforce the need for multimodal programs combining aerobic and strength training at adequate intensity and progression [4], together with strategies to preserve lean body mass—particularly when energy restriction is applied [18]. Tailoring the intervention to baseline status (obesity, physical inactivity, fatigue or endocrine therapy) and using objective measurement of physical activity are key to optimizing efficacy, as discussed by Greenlee et al. [2] and Zhao et al. [37]. These data support the structured integration of supervised exercise and nutritional counseling into routine oncological follow-up, with clearly defined goals for body composition and behavioral adherence, in keeping with the 2022 American Cancer Society guideline [33].

Although exercise prescriptions of 150–200 min per week are commonly recommended and were associated with beneficial outcomes in several included studies, their implementation may be challenging in real-world settings, particularly for survivors with limited free time, demanding work schedules, caregiving responsibilities, treatment-related fatigue, or cardiometabolic and musculoskeletal comorbidities. In resource-limited settings, restricted access to supervised facilities and transport costs constitute additional structural barriers that most included trials do not adequately capture. Implementation-oriented research should therefore evaluate whether accumulated shorter bouts (10–15 min), home-based protocols with remote supervision, and community-based services can reproduce supervised trial effects while improving accessibility and equity, in line with the implementation framework above [36].

### 4.8. Strengths and Limitations

The results should be interpreted in light of several limitations. First, methodological quality varied considerably across the included studies: 11 were randomized controlled trials while four were single-arm pre–post, pilot or cohort designs, which by their nature provide lower certainty of evidence and cannot rule out regression to the mean, Hawthorne effect or concomitant treatments. The Discussion should therefore be read with this evidence hierarchy in mind: claims based on Dieli-Conwright et al. [25] or Brown et al. [18] carry greater weight than claims derived from single-arm studies such as Travier et al. [20] or Fabian et al. [26]. Second, marked clinical and methodological heterogeneity in intervention type, delivery mode, intensity, duration and outcome measures prevented a meta-analysis and limits direct comparability between trials. Third, many trials had moderate sample sizes and relatively short follow-up; the results therefore reflect short- to medium-term effects and do not allow firm conclusions on long-term sustainability. Fourth, several digital interventions relied primarily on self-reported dietary and physical activity data, which are susceptible to recall and social desirability bias; studies that did not incorporate objective measurements such as accelerometry, validated food records or wearable monitors must be interpreted with extra caution, and their effect sizes are likely to be inflated compared with biomarker-validated outcomes. Fifth, a possible publication bias must be acknowledged: positive intervention studies are more likely to be published than null or negative findings, which may inflate the aggregate estimate of effect; we could not formally evaluate this risk because of the small number and heterogeneity of the included trials. Finally, although several metabolic and inflammatory biomarkers improved, none of the included studies were designed to assess hard oncological outcomes (recurrence or survival); any inference on prognosis remains indirect and requires confirmation in larger, longer-term trials.

Among the strengths of this review, the inclusion of heterogeneous lifestyle interventions, delivery formats and survivor populations reflects the real-world complexity of survivorship care and increases the external validity of the synthesized conclusions, even though it limits direct quantitative pooling. The systematic assessment of risk of bias with RoB 2/ROBINS-I [14,15] and certainty of evidence with GRADE [16] for each outcome domain, the explicit subgroup analysis by intervention modality (multimodal supervised, exercise alone, diet alone, digital), and the transparent reporting of effect sizes alongside qualitative interpretation strengthen the transparency and methodological robustness of the synthesis.

### 4.9. Future Research Directions

A central research priority is to establish whether the metabolic and behavioral improvements observed in this review translate into hard oncological outcomes. The current evidence base supports that lifestyle interventions help survivors feel better—through improvements in body composition, biomarkers, fatigue, and quality-of-life domains—but whether these gains reduce recurrence or extend survival remains unproven. Bridging this gap should be a central goal of the next generation of exercise oncology trials, requiring multicenter designs, adequate statistical power, prolonged follow-up, and hard oncological endpoints such as recurrence-free or overall survival.

Building on the present synthesis, we propose the development and prospective evaluation of a standardized “supportive-care bundle” for breast cancer survivors. Such a bundle could integrate: (i) supervised aerobic plus resistance exercise of 150–200 min/week of MVPA, with progressive escalation when feasible; (ii) Mediterranean-style nutritional counseling delivered by qualified dieticians, with emphasis on energy adequacy and micronutrient sufficiency; (iii) objective monitoring of physical activity through accelerometry or wearable devices; (iv) behavioral and psychological support tailored to baseline fatigue, mood and comorbidities; and (v) hybrid delivery formats to accommodate work, caregiving and accessibility barriers. Evaluating this bundle in pragmatic, multicenter trials with hard oncological endpoints would offer a reproducible framework for comparability across studies and a translational pathway from intermediate biomarkers to clinical benefit, in line with the consensus statements [32,33,36].

Mechanistic studies that integrate metabolomic and nutrigenomic approaches could also explore whether inter-individual variability in metabolic pathways relevant to estrogen metabolism modulates the response to lifestyle interventions; this remains a hypothesis-generating direction, as none of these mechanisms were assessed in the studies included in this review.

## 5. Conclusions

This systematic review indicates that multimodal interventions combining structured nutritional counseling and planned physical exercise are feasible and effective for improving body composition, cardiometabolic profile, and quality of life in breast cancer survivors. The most robust effects are observed when guided energy restriction is integrated with combined aerobic and strength training, performed at sufficient intensity and under adequate supervision. The evidence supports improvements in total and visceral adiposity, as well as in metabolic biomarkers related to insulin, adipokines, and cardiometabolic risk. However, it must be explicitly acknowledged that the current evidence does not allow us to determine whether these improvements in body composition and metabolic biomarkers translate into a reduction in the risk of recurrence or mortality. The studies included in this review were not designed to assess these hard oncological outcomes, and any inference regarding prognosis remains indirect. Methodological heterogeneity and limited follow-up further preclude firm conclusions in this regard. Multicenter trials with greater statistical power, prolonged follow-up, and recurrence or survival as primary endpoints are required to establish whether the observed metabolic and functional benefits confer a meaningful reduction in oncological risk.

## Figures and Tables

**Table 4:** Summary of effects by intervention type on body composition (with GRADE certainty).

Intervention Type	Outcomes	Magnitude of Effect	Consistency	GRADE Certainty	References (Author, Year)	Observations
Multimodal supervised (diet + exercise)	Body weight, BMI, total fat, VAT, SAT, waist circumference	↓ weight 5–8%; ↓ VAT up to 19–29%; ↓ SAT and waist circumference; ↓ total fat mass	High	Moderate	Brown 2021 [18]; Arikawa 2018 [23]; Travier 2018 [20]; Ruiz-Vozmediano 2020 [31]; Pistelli 2021 [22]; Fabian 2021 [26]; Suzuki 2020 [19]; Buckland 2019 [24]	Greatest effects when ≥6 months and direct supervision
Exercise alone (combined aerobic + resistance)	Fat mass, VAT, SAT, sarcopenic obesity	↓ VAT, SAT and total fat; preserved/improved lean mass	Moderate	Moderate	Dieli-Conwright 2018 [25]; García-Unciti 2023 [27]	Effective even without dietary restriction; preserves lean mass
Diet alone	Body weight, lean body mass	↓ weight (modest); concomitant ↓ lean body mass ≈ 0.8–1.2 kg/year	Low (single arm of multi-arm trial)	Low	Brown 2021 [18] (diet-only arm)	Risk of muscle mass loss without exercise
Digital/eHealth	Body weight, BMI, SAT, TAT	Heterogeneous: no change in BMI/weight (Greenlee, Holtdirk); ↓ SAT and TAT [21]	Low	Very low	Greenlee 2024 [2]; Holtdirk 2021 [29]; Sturgeon 2018 [21]	Improves dietary behaviors but limited effect on objective body composition

BMI: body mass index; LM: lean mass; SAT: subcutaneous adipose tissue; TAT: total adipose tissue; VAT: visceral adipose tissue. GRADE certainty: high; moderate; low; very low; ↓: Decrease in value compared to the initial value.

**Table 5:** Summary of effects by intervention type on metabolic biomarkers and adipokines (with GRADE certainty).

Intervention Type	Biomarkers	Magnitude of Effect	Consistency	GRADE Certainty	References	Observations
Multimodal supervised (diet + exercise)	Fasting insulin, HOMA-IR, lipid profile (TG, total cholesterol, LDL-C), leptin, adiponectin	↓ insulin (~−15%); ↓ HOMA-IR (~−13%); ↓ TG, total cholesterol and LDL-C; ↓ leptin; ↑ adiponectin [26]	High	Moderate	Buckland 2019 [24]; Arikawa 2018 [23]; Reis 2021 [30]; Ruiz-Vozmediano 2020 [31]; Travier 2018 [20]; Fabian 2021 [26]; Suzuki 2020 [19]; Pistelli 2021 [22]	Broadest and most wide-ranging biomarker changes
Exercise alone (supervised combined)	Insulin, IGF-1, leptin, adiponectin, MetS z-score	↓ insulin, IGF-1 and leptin; ↑ adiponectin; ↓ overall MetS z-score; effects maintained at 3 months	High (single high-quality RCT)	Moderate	Dieli-Conwright 2018 [25]	Effect independent of caloric restriction
Digital/eHealth	Insulin, SAT, IL-1β, IL-6, IL-8, TNF-α	↓ insulin associated with ↓ SAT (r = 0.33); no significant change in IL-1β, IL-6, IL-8; ↓ TNF-α within-group only	Low	Very low	Sturgeon 2018 [21]	Insufficient body composition change to modify systemic inflammation

Abbreviations: HOMA-IR: homeostatic model assessment of insulin resistance; IGF-1: insulin-like growth factor-1; IL-1β/IL-6/IL-8: interleukin-1β/-6/-8; LDL-C: low-density lipoprotein cholesterol; MetS: metabolic syndrome; SAT: subcutaneous adipose tissue; TG: triglycerides; TNF-α: tumor necrosis factor alpha; ↓: Decrease in value compared to the initial value; ↑: Increase in value compared to the initial value.

**Table 6:** Summary of effects by intervention type on functional capacity and fatigue (with GRADE certainty).

Intervention Type	Outcomes	Magnitude of Effect	Consistency	GRADE Certainty	References	Observations
Multimodal supervised (diet + exercise)	Functional capacity (6MWT, VO_2_peak, METs, muscle strength); fatigue (EORTC QLQ-C30, CFS, BFI)	↑ functional scores (Δ +25–34 pts EORTC; ↑ 6MWT completion 17%→73%); ↓ fatigue from −14.9% up to >80%	High in supervised programs	Moderate	Arikawa 2018 [23]; Ghavami 2017 [28]; Ruiz-Vozmediano 2020 [31]; Suzuki 2020 [19]; Pistelli 2021 [22]; Fabian 2021 [26]	Larger effects with supervised in-person delivery
Exercise alone or with mind–body component	Cardiorespiratory fitness, muscle strength, fatigue	↑ METs, ↑ VO_2_peak, ↑ strength; consistent ↓ fatigue; mind–body adjuncts reinforce fatigue reduction	Moderate	Moderate	Dieli-Conwright 2018 [25]; García-Unciti 2023 [27]; Ruiz-Vozmediano 2020 [31]; Suzuki 2020 [19]	Mind–body adjuncts may potentiate fatigue improvement
Digital/eHealth	PROMIS physical function, fatigue scales	Holtdirk [29]: QoL d = 0.27 (significant); fatigue d = 0.23 (secondary outcome); PA d = 0.30 but not significant after Bonferroni adjustment. Greenlee [2]: no significant effect on PROMIS function (adjusted difference −0.56; 95% CI −1.27 to 0.15) or MVPA	Low	Very low	Greenlee 2024 [2]; Holtdirk 2021 [29]	No effect on objectively measured MVPA

Abbreviations: 6MWT: six-minute walk test; BFI: Brief Fatigue Inventory; CFS: Cancer Fatigue Scale; EORTC QLQ-C30: European Organization for Research and Treatment of Cancer Quality of Life Questionnaire-Core 30; METs: metabolic equivalents of task; MVPA: moderate-to-vigorous physical activity; PROMIS: Patient-Reported Outcomes Measurement Information System; VO_2_peak: peak oxygen uptake; ↓: Decrease in value compared to the initial value; ↑: Increase in value compared to the initial value.

**Table 7:** Summary of effects by intervention type on quality of life and psychological well-being (with GRADE certainty).

Intervention Type	Outcomes	Magnitude of Effect	Consistency	GRADE Certainty	References	Observations
Multimodal supervised (diet + exercise ± mindfulness)	Global QoL (EORTC QLQ-C30); HADS (anxiety/depression); psychological distress (K6); self-efficacy (SES)	↑ global QoL +14 to +34 pts; ↑ physical, role and social functioning; ↓ anxiety, depression, fatigue, pain and nausea/vomiting	Moderate–high	Moderate	Ghavami 2017 [28]; Ruiz-Vozmediano 2020 [31]; Suzuki 2020 [19]; Pistelli 2021 [22]	Strongest effects in programs ≥ 6 months with group component
Digital eHealth	HRQoL, depression, anxiety, fatigue	Small to moderate effects (d ≈ 0.27); improvements limited to specific psychological domains	Low	Low	Greenlee 2024 [2]; Holtdirk 2021 [29]	No significant changes in functional QoL or anxiety

Abbreviations: EORTC QLQ-C30: European Organization for Research and Treatment of Cancer Quality of Life Questionnaire-Core 30; HADS: Hospital Anxiety and Depression Scale; HRQoL: health-related quality of life; K6: Kessler 6-item Psychological Distress Scale; QoL: quality of life; SES: Self-Efficacy Scale for Cancer Patients; ↓: Decrease in value compared to the initial value; ↑: Increase in value compared to the initial value.

**Table 8:** Summary of effects by intervention type on dietary habits (with GRADE certainty).

Intervention Type	Outcomes	Magnitude of Effect	Consistency	GRADE Certainty	References	Observations
Multimodal supervised (diet + exercise)	Energy intake, fat intake, micronutrients, Mediterranean diet adherence	↓ energy ≈ 170 kcal/day (15–20%); ↓ saturated and total fat (10–31%); ↑ carotenoids (+15.1%) and B vitamins (+5–9%); ↑ MD adherence (+1.57 pts)	High	Moderate	Buckland 2019 [24]; Reis 2021 [30]; Ruiz-Vozmediano 2020 [31]; Travier 2018 [20]; Arikawa 2018 [23]; Pistelli 2021 [22]	Most reproducible and biologically validated dietary changes
Diet alone or with light exercise advice	Mediterranean diet adherence, micronutrient intake	Modest or non-significant changes; only ≈19% high MD adherence; persistent deficits in Ca, Zn, folate and vitamins D, A and E	Low	Very low	García-Unciti 2023 [27]; Brown 2021 [18] (diet-only arm)	Diet without structured counseling is insufficient
Digital/eHealth	Fruit and vegetable intake, energy intake, diet quality	↑ F&V +1.1–1.5 servings/day; moderate improvement (d ≈ 0.36); heterogeneous results	Moderate	Low	Greenlee 2024 [2]; Holtdirk 2021 [29]; Sturgeon 2018 [21]	Effective for behavioral change; limited by self-reporting

Abbreviations: F&V: fruit and vegetables; MD: Mediterranean diet; ↓: Decrease in value compared to the initial value; ↑: Increase in value compared to the initial value.

**Table 9:** Summary of effects by intervention type on inflammatory and immune markers (with GRADE certainty).

Intervention Type	Biomarkers	Magnitude of Effect	Consistency	GRADE Certainty	References	Observations
Multimodal supervised (diet + exercise)	Leptin, F2-isoprostanes, CRP, IL-6, adiponectin, IGF-1 [23]; adiponectin:leptin ratio [26]	↓ leptin (47 → 19 → 26.2 ng/mL); ↓ F2-isoprostanes (71.5 → 50 ng/mL); ↓ CRP [23]; IL-6 and adiponectin unchanged; IGF-1 paradoxically increased with weight loss. ↑ adiponectin:leptin ratio 0.77 → 1.08 [26]	Low–moderate	Low	Arikawa 2018 [23]; Fabian 2021 [26]	Anti-inflammatory trend; few studies measured these biomarkers
Exercise alone (supervised combined)	Insulin, IGF-1, leptin, adiponectin, IL-6, IL-8, TNF-α, hsCRP	↓ insulin, IGF-1 and leptin; ↑ adiponectin; ↓ IL-6, IL-8, TNF-α and hsCRP (all *p* < 0.001); maintained at 3 months	High (single high-quality RCT)	Moderate	Dieli-Conwright 2018 [25]	Broadest inflammatory panel; effect independent of caloric restriction
Digital/eHealth	Insulin, IL-1β, IL-6, IL-8, TNF-α	↓ insulin associated with ↓ SAT; ↓ TNF-α within-group only; no significant changes in IL-1β, IL-6, IL-8	Low	Very low	Sturgeon 2018 [21]	Magnitude of body composition change insufficient to modulate systemic inflammation

Abbreviations: CRP: *C*-reactive protein; hsCRP: high-sensitivity *C*-reactive protein; IGF-1: insulin-like growth factor-1; IL-1β/IL-6/IL-8: interleukin-1β/-6/-8; SAT: subcutaneous adipose tissue; TNF-α: tumor necrosis factor alpha; ↓: Decrease in value compared to the initial value; ↑: Increase in value compared to the initial value.

## Data Availability

The original data supporting the findings of this study are included in the article, and any additional information may be obtained from the corresponding author upon reasonable request.

## Supplementary Materials

The following supporting information can be downloaded at https://www.mdpi.com/article/10.3390/nu18111815/s1, Table S1: Search strategy in Scopus; Table S2: Search strategy in Web of Science; Table S3: Methodological quality assessment process using the RoB 2.0 tool; Table S4: Methodological quality assessment process using the ROBINS-I tool. Table S5: GRADE Domain. Assessment and justification.

## Abbreviations

The following abbreviations are used in this manuscript:

MVPA	Moderate-to-vigorous physical activity
YES	Yale Exercise and Survivorship
BMI	Body mass index
F &V	Fruit and vegetables
DQI	Diet quality index
VAT	Visceral adipose tissue
HOMA-IR	Insulin resistance
ACSM/ACS	American College of Sports Medicine/American Cancer Society
IGF-1	Insulin-like growth factor-1
IL-6	Interleukin-6
IL-10	Interleukin-10
CRP	*C*-reactive protein
HRQoL	Health-related quality of life
IGFBP-3	Insulin-like growth factor binding protein 3
FACT-B	Functional Assessment of Cancer Therapy—Breast
